# The impact of physical activity on cognitive, behavioral, and academic performance in children with ADHD: a systematic review

**DOI:** 10.1590/1980-5764-DN-2025-0339

**Published:** 2025-12-01

**Authors:** Gabriel de Brito Porfirio, Mateus do Carmo Bardella, Merlyn Mércia Oliani, Camila de Moraes

**Affiliations:** 1 Universidade de São Paulo, Faculdade de Educação Física e Esporte de Ribeirão Preto, Ribeirão Preto SP, Brazil.; 2 Centro Universitário Estácio Ribeirão, Programa de Educação Física, Ribeirão Preto SP, Brazil.

**Keywords:** Neurodevelopmental Disorders, Exercise, Behavior, Cognition, Child, Transtornos do Neurodesenvolvimento, Exercício Físico, Comportamento, Cognição, Criança

## Abstract

Attention Deficit/Hyperactivity Disorder (ADHD) is a neurodevelopmental disorder affecting many children worldwide. They face challenges in multiple cognitive, behavioral, and emotional functions. It is well established that engaging in physical exercise enhances physical capacities and is associated with improved cognitive performance in children.

**Objective:**

This systematic review aimed to examine the role of physical activity as a non-pharmacological approach for enhancing the cognitive, and consequently, academic and behavioral, performance in children with ADHD.

**Methods:**

Clinical trials from the last 10 years were searched in the PubMed, LILACS, and Cochrane databases, with 11 studies included in this review.

**Results:**

All studies demonstrated improvements in cognitive domains, including memory, attention, and inhibition, as well as behavioral benefits.

**Conclusion:**

Published data indicate that physical activity positively impacts the cognitive, academic, and behavioral performance of children with ADHD.

## RESUMO

O Transtorno de Déficit de Atenção/Hiperatividade (TDAH) é um distúrbio do neurodesenvolvimento que acomete muitas crianças ao redor do mundo, gerando desafios em múltiplas funções cognitivas, comportamentais e emocionais. Sabe-se que a prática de exercício físico melhora as capacidades físicas e está associada ao aumento do desempenho cognitivo em crianças.

**Objetivo:**

Examinar o papel da atividade física como abordagem não farmacológica para a melhoria do desempenho cognitivo e, consequentemente, acadêmico e comportamental em crianças com TDAH.

**Métodos:**

Ensaios clínicos dos últimos 10 anos foram pesquisados nas bases de dados PubMed, LILACS e Cochrane, sendo incluídos 11 estudos nesta revisão.

**Resultados:**

Todos os estudos demonstraram melhorias em domínios cognitivos, incluindo memória, atenção e inibição, além de benefícios comportamentais.

**Conclusão:**

Os dados publicados indicam que a atividade física impacta positivamente o desempenho cognitivo, acadêmico e comportamental de crianças com TDAH.

## INTRODUCTION

 Cognition includes memory, attention, perception, language, decision-making, and visuospatial and executive functions^1^. Executive functions are a set of cognitive processes that direct actions and behaviors essential for learning and daily performance. These functions contribute to task monitoring and regulation, extending beyond the cognitive domain to encompass socioemotional and behavioral aspects of human functioning^2^. 

 There is a consensus that three fundamental executive functions exist: inhibition, working memory, and cognitive flexibility^3^. Inhibition allows children to control behavior, attention, thoughts, and emotions to take the most appropriate actions, suppressing strong internal predisposition or blocking habitual or inappropriate responses. Working memory refers to the short-term storage and manipulation of information needed for practical taks. Cognitive flexibility enables children to shift attention between task demands and modify approaches to problem-solving, adapting to new requirements, rules, or priorities^3^. 

 Worldwide, attention deficit hyperactivity disorder (ADHD) affects 3 to 8% of children and adolescents. Prevalence rates are higher in children under 12 than in adolescents, and the disorder is more common in boys than in girls^4^. In Brazil, ADHD prevalence aligns with global estimates, affecting approximately 7.6% of children and adolescents aged 6 to 17 years^5^. 

 According to the Diagnostic and Statistical Manual of Mental Disorders^6^, ADHD is a neurodevelopmental disorder characterized by deficits in inattention, disorganization, and/or hyperactivity-impulsivity, resulting in impairments in social, cognitive/academic, and occupational functioning. Inattention is characterized by difficulty maintaining focus, lack of persistence, a tendency to stay away from tasks, and disorganization^6^. Hyperactivity is defined as excessive or inappropriate motor activity, fidgeting, tapping, or excessive talking^6^. Impulsivity reflects a desire for immediate rewards or an inability to delay gratification^6^. 

 Children with ADHD often struggle to perform adequately in various cognitive domains, including problem-solving, planning, orientation, cognitive flexibility, sustained attention, response inhibition, and working memory^5^. Between half and two-thirds of school-age children with ADHD also present co-occurring psychiatric and developmental disorders^4^. In the long term, these difficulties can affect academic performance, interpersonal relationships, and self-esteem^4,5^. 

 The signs of ADHD usually become more evident when children’s responsibilities and independence increase, for example, at the beginning of individual activities in a school setting or when organizing tasks without parental supervision^5^. Consequently, the disorder is often identified and treated in the early elementary school years, before age 12^4,6^. 

 Risk factors commonly associated with ADHD include certain temperamental aspects, such as lower levels of behavioral inhibition and negative affectivity; environmental elements, including very low birth weight (less than 1.5 kg, associated with a 2- to 3-fold higher risk), maternal exposure to toxins from smoking, alcoholism, lead-contaminated water, etc.; and genetic and physiological factors, with heritability being more likely when first-degree biological relatives have the disorder^6^. Although early childhood family interactions do not cause ADHD, they can influence the development of secondary conduct problems, acting as modifiers of the disorder’s course^6^. 

 A diagnostic assessment is conducted by a specialized physician, such as a psychiatrist, pediatrician, or other qualified healthcare professional, who must have appropriate qualifications, including training and experience in ADHD^5^. The primary diagnostic classification systems used as reference are:

- the International Statistical Classification of Diseases and Related Health Problems, 1^st^ edition^7^, corresponding to code F90, of the World Health Organization (WHO);
- The Diagnostic and Statistical Manual of Mental Disorders, 5^th^ edition^6^.

These classification systems are similar, although the American Psychiatric Association (APA) criteria are more up-to-date. 

 Regarding treatment, medications such as methylphenidate, whose mechanism of action involves stimulating alpha- and beta-adrenergic receptors and releasing dopamine and norepinephrine from synaptic terminals^8^, as well as lisdexamfetamine, which acts by blocking dopamine reuptake and stimulating its production and that of norepinephrine^9^, can be used. Non-pharmacological treatment may include multidisciplinary approaches aimed at improving symptoms, executive control, and social and occupational functioning associated with this complex condition^5^. Cognitive-behavioral therapy employs techniques that enable the patient (child or adult) to restructure beliefs based on more adaptive perspectives, suppress or mitigate conditioned and maladaptive behaviors, and modify thoughts, emotions, and sensations. Cognitive techniques include cognitive restructuring, problem-solving, and inner dialogue, while behavioral techniques include self-monitoring and self-assessment, a reward system, and response cost^5,10^. 

 Another approach that may be included in the therapeutic strategy is physical activity and exercise, including sports and exergames, which have been considered fundamentally important for the effective treatment of children with ADHD. These activities provide numerous cognitive benefits^11^, as well as behavioral benefits, including reductions in symptoms of depression and anxiety^12,13^. 

 This systematic review aimed to assess the impact of physical activity and exercise as non-pharmacological interventions to improve cognitive, academic, and behavioral performance in children with ADHD. 

## METHODS

 The methodology was developed in accordance with the PRISMA guidelines^14^, and the PICO acronym, a strategic research tool^15^, guided the study search. 

### Database search strategy

 The PubMed, LILACS, and Cochrane databases were used. For PubMed, the search terms and Boolean operators were: ("child" OR "preschool" OR "children" OR "elementary schoolchildren" OR "ADHD" OR "children with ADHD") AND ("exercise" OR "games" OR "exergaming" OR "recreational" OR "play") AND ("cognition" OR "executive function" OR "academic success" OR "academic achievement"). For LILACS and Cochrane, the terms were: ("children with ADHD") AND ("exercise") AND ("cognition"). 

### Selection criteria

 The selection criteria included randomized controlled trials published in the last 10 years (2014–2024), with samples composed of children or preschoolers with ADHD, interventions involving physical activity/exercise (including sports, play, games, or exergames), a control/comparison group maintaining sedentary behavior, and outcomes aimed at improving cognitive, academic, and behavioral domains, using any type of validated test to measure cognitive domains. Studies were excluded if there was a lack of supervision during training, insufficient description of the physical activity/exercise protocol, a control group that received training, populations with conditions other than ADHD, or interventions that included complementary factors in addition to physical exercise. 

### Data collection

**Figure 1:**
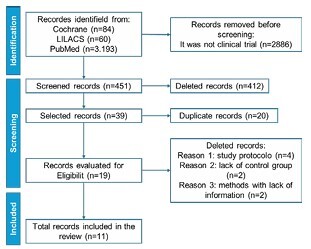
PRISMA flow diagram for identifying studies through database and registration.

 The extracted data on the sample included sample size, gender, age, and mean age. For the intervention group, information was collected on the duration of the supervised intervention (in weeks), the weekly frequency of activities/exercises, the types of activities practiced, and the cognitive tests applied. The outcomes collected for both groups included descriptive result in the cognitive, academic, and behavioral domains (the latter two when reported). Following the search and study selection process, 11 clinical trials were included, featuring multiple types of activities and physical exercises as interventions, along with cognitive assessments. Details of the study selection are presented in Figure 1. 

## RESULTS

 Literature findings suggest that physical exercise can positively impact various domains of cognitive function in children. Ziereis and Jansen^16^ reported improved memory performance in children following interventions involving multiple exercises and sports. Other studies corroborated these improvements through recreational swimming^13^, aerobic and neurocognitive exercises^17^, and judo^18^. 

 Inhibition reaction time was also positively affected. According to Pan et al.^12^, improvements in inhibition reaction time were observed after table tennis intervention, similar to results reported by Chang et al.^19^ using the same sport. Significant improvements in these domains were also demonstrated with other strategies, including different modalities of aerobic exercise^17,20^, exergames^21,22^ and recreational swimming^13,23^. The domain of attention improved after playing table tennis in both real and simulated environments^12,19^, as well as following swimming practice^11,23^. Finally, task switching^21,22^ and cognitive flexibility^11,17^ were also enhanced. 

 Only one study assessing academic outcomes identified improvements in reading comprehension and mathematical reasoning among children who participated in recreational swimming^13^. In terms of behavioral outcomes, decreases were observed in somatic complaints^13^, symptoms of anxiety/depression^11-13^, and withdrawn or aggressive behavior^11,12,20,23^. Table 1 provides a detailed description of the studies that evaluated the influence of physical activity on different cognitive domains. 

**Table 1:** Summary of the primary outcomes of this systematic review.

References	Sample	Intervention	Cognitive assessment	Cognitive/behavioral outcomes
Silva et al.^11^	N: 35 (ND♂/ND♀) Age: ND (11–14)	W: 8 / WF: 2x / D: 45’ Swimming	TMT. Cancellation Attention Test (CAT). Children’s Depression Inventory (CDI). Perceived Stress Scale (PSS 14).	↑ Cognitive Flexibility and Selective Attention. ↓ Depression and Stress.
Pan et al.^12^	N: 32 (ND♂) Age: ND (6–12)	W: 12 / WF: 2x / D: 70’ Table tennis	Stroop-color-word test Child Behavior Checklist.	↑ Selective Attention and Response Inhibition. ↓ Anxiety/depression, Aggressive Behavior, etc.
Hattabi et al.^13^	N: 40 (35♂/5♀) Age: ND (9–12)	W: 12 / WF: 3x / D: 90’ Recreational Swimming	Hayling Junior Test. Child Behavior Checklist. Academic Performance (test of reading comprehension, mathematics, and general average).	↑ Response Inhibition and Notes. ↓ Somatic Complaints, Anxiety/Depression, Thinking Problems, and Withdrawal.
Ziereis and Jansen^16^	N: 39 (11♂/32♀) Age: 9.4 (7–12)	W: 12 / WF: 1x / D: 60’ Exercises and Various Sports	Digit Range Task. Letter and Number Sequencing Task. Corsi Block Beat Test.	↑ Verbal Working Memory. ↔ Processing Update.
Liang et al.^17^	N: 80 (ND♂/ND♀) Age: 8.4 (6–12)	W: 12 / WF: 3x / D: 60’ Aerobic Exercises and Neurocognitive Exercises	Flanker task Tower of London Test TMT	↑ Response Inhibition Reaction Time, Working Memory and Cognitive Flexibility.
Ludyga et al.^18^	N: 57 (ND♂/ ND♀) Age: ND (8–12)	W: 12 / WF: 2x / D: 60’ Judo	Existing script of a modified (computerized) change detection paradigm	↑ Operational Memory.
Chang et al.^19^	N: 48 (ND♂/ND♀) Age: ND (ND)*	W: 12 / WF: 3x / D: 60’ Real Table Tennis (IG I) and Simulated (IG II)	Stroop Color Test. Stroop-color-word Test. Wisconsin Card Sorting Test.	↑ Selective Attention and Response Inhibition (Most Significant Expression in Real Table Tennis Intervention).
Memarmoghaddam et al.^20^	N: 40 (ND♂) Age: ND (7–11)	W: 8 / WF: 3x / D: 90’ Various Aerobic Exercises with Balls, Rackets, etc.	Stroop test. Go-no-go test. Child behavior checklist.	↑ Response Inhibition. ↑ Behavior.
Benzing et al.^21^	N: 51 (ND♂/ND♀) Age: 10.4 (8–12)	W: ND / WF: 1x / D: 15’ Exergame	Modified Flanker Task. Modified Color Extension Retroactive Task.	↑ Response Inhibition, Reaction Time, and Task Switching. ↔ Visual Working Memory Performance.
Benzing and Schimidt^22^	N: 51 Age: 10.4 (8–12)	W: 8 / WF: 3x / D: 30’ Exergame	Modified Simon Task. Modified Flanker Task. Retroactive Color Extension Task.	↑ Response Inhibition, Reaction Time, and Task Switching.
Hattabi et al.^23^	N: 40 (ND♂/ND♀) Age: ND (8–12)	W: 12 / WF: 3x / D: 90’ Recreational Swimming	Rey Osterrieth Complex Figure. Stroop test. Hayling Junior Test.	↑ Memory Accuracy, Selective Attention, and Response Inhibition.

Abbreviations: N, sample size; ♂, male sex; ♀, female; ND, Not described; Age represented by mean and range (minimum and maximum); W, weeks; WF, weekly frequency; D, the duration of each session in minutes; ↑, improvement; ↓, decrease; ↔, no significant change; IG I, intervention group I; IG II, intervention group II; TMT, Trail Making Test; *, the study did not provide age values but indicated that the participants were children from the 1st to the 6th school year; -, the data was not presented.

## DISCUSSION

 This systematic review aimed to investigate the impact of physical activity and exercise on cognitive performance in children with ADHD. Eleven clinical trials were reviewed, involving multiple modalities of physical activity and exercise as interventions, alongside cognitive assessments. The results indicated cognitive benefits in the following domains, as evaluated using standard tests: memory, attention, inhibition, task switching, and cognitive flexibility. Improvements were also observed in academic outcomes, as assessed by reading comprehension and mathematics tests, as well as behavioral modifications, including reductions in anxiety and depressive symptoms. For a detailed description, see Table 1. 

 The interventions were predominantly aerobic and delivered in a fun and dynamic manner. Although they varied in both practice and resources used, the interventions were generally replicable and accessible. Notably, exergames were highlighted as particularly interesting, as they allow the practice of cognitively challenging activities, including non-automated movements that demand considerable coordination and speed of action^21^. 

 Regarding interventions in general, two critical aspects were identified: the diversity of physical activities and their implementation with children. Physical activity sessions ranged from 15 to 90 minutes, with two interventions lasting 15 and 30 minutes^21,22^ using exergames. These shorter sessions demonstrated significant acute effects on response inhibition, reaction time, and task switching in children with ADHD, suggesting that even brief, 15-minute sessions at moderate to vigorous intensity can enhance certain cognitive functions^21,24^. These findings expand the understanding of the role of volume and intensity in selecting suitable physical activities and exercises. Previous reviews demonstrated a positive link between physical activity and both cognitive performance and academic achievement in childhood populations^25,26^. However, similar to our review, there is no consensus regarding the necessary duration of intervention. The studies included in the present review featured protocols lasting 8 to 12 weeks, with a weekly frequency of 2 to 3 sessions, and variations in the duration of training sessions, as mentioned above. 

 The second aspect underscores the importance of considering the professional’s conduct and interaction with the children to ensure the successful implementation of the interventions. For instance, in the study by Benzing et al.^22^, the absence of incentives, weekly communication, and feedback on training frequency resulted in a relatively high dropout rate, with seven children withdrawing before the post-test. In contrast, the study by Pan et al.^12^ provided individualized instruction, immediate positive reinforcement, and feedback on the accuracy of each child’s movements after each training session, resulting in zero dropouts and demonstrating adequate program adherence. 

 Cognitive assessment varies across domains, with multiple tests available for each domain, and results can be reported as absolute scores or percentiles. Consequently, cognitive outcomes are presented descriptively in Table 1. For example, working memory (including verbal, visual, and visuospatial components) was assessed using the digit span task, letter-number sequencing task, Corsi block-tapping test, modified change detection task, Tower of London task, backward color-figure extension task, and Rey-Osterrieth complex figure. Inhibition was assessed using the Simon task, Stroop test, Junior Hayling test, Wisconsin Card Sorting Test, and go/no-go test. Task switching was assessed with the Flanker task, cognitive flexibility with the Trail Making Test, and selective attention with a cancellation attention test. 

 Most of these tests were repeatedly used across studies, with modifications to the Simon task, Flanker task, and change detection task reported in only three studies^18,21,22^. Regarding behavioral assessment, the Child Behavior Checklist (CBCL), academic performance tests (including reading comprehension, mathematics, and overall average), the Child Depression Inventory (CDI), and the Perceived Stress Scale (PSS-14) were used. In addition, handwriting ability was improved in the study by Chang et al.^19^. This heterogeneity of assessments is essential, as it enables evaluation through various methods and approaches, considering accessibility and replicability. 

 All studies demonstrated cognitive improvements in children with ADHD, reinforcing the importance of physical activity and exercise in this context. Notably, the benefits extended beyond the immediate post-program period, influencing children’s daily lives, particularly in the academic domain, with improved performance in reading comprehension and mathematical ability^13^. This aspect has considerable potential for further exploration, as demonstrated by Pagani et al.^27^, which showed that sports participation in childhood is associated with higher indicators of academic success at the end of adolescence, potentially enhancing opportunities in adult life, as well as promoting behavioral changes^12,13,20^. Physical activity and exercise, when appropriately developed by a professional, can become an effective strategy and should be part of the therapy for children with ADHD. Interventions in the form of games, for example, can effectively maximize performance in children with ADHD^28^. Working in small or large groups, ensuring adequate professional supervision, and implementing an effective behavior management system can help reduce social problems and foster friendships among participants^12^. In the study by Pan et al.^12^, some parents wrote thank-you notes, indicating that their children enjoyed the sessions and looked forward to meeting the coaches and other participants each week. Parents also reported that the program provided opportunities to develop social networks with other family members. When activities are engaging, playful, responsible, and challenging, with consistent interactions and feedback, the resulting improvements in cognitive function, academic performance, and behavior are more pronounced and enduring. 

 Physical activity and exercise have a significantly positive impact on the cognitive, behavioral, and academic performance of children with ADHD. The diversity of intervention protocols, including aerobic activities and exergames, demonstrates that different approaches are effective if conducted in a structured manner and tailored to the needs of the children. Physical activity may affect cognition through multiple mechanisms. Brain-derived neurotrophic factor (BDNF) appears particularly important, as it promotes synaptogenesis — a process directly related to cognitive functions^29,30^. Reycraft et al.^31^ showed that BDNF levels increase following aerobic exercise at different intensities. Physical exercise can also induce cerebral angiogenesis, thereby improving blood flow and facilitating the delivery of oxygen and essential nutrients, which contribute to enhanced cognitive function^32^. 

 The mechanisms underlying physical exercise-induced cerebral and cognitive adaptations have been studied in animal models. Marlatt et al.^33^ demonstrated that in female rats, regular aerobic exercise at the onset and during middle age could maintain brain function. Physical exercise increased both BDNF levels and neurogenesis and improved spatial memory retention. Van Praag et al.^34^ demonstrated that aged rats with free access to physical activity exhibited increased neurogenesis and improved memory compared to sedentary aged rats. Mirochnic et al.^35^ showed that transgenic mice with features of Alzheimer disease (APP23 mice) exhibited increased hippocampal neurogenic activity after engaging in physical activity at an advanced age. Horowitz et al.^36^ investigated the effects of exercise-induced blood-borne factors on neurogenesis and cognitive function in aged mice, showing that the infusion of plasma from aged exercising mice into sedentary aged mice resulted in significant improvements in neural regeneration and cognition. The enzyme Gpld1, produced in the liver and elevated after physical activity, was identified as a key mediator of these effects. One limitation of the current study is the challenge of applying findings from animal models to human beings. While animal research provides valuable insights into the neurobiological mechanisms linking physical activity and cognition, species differences restrict the direct application of these results to humans. Therefore, the findings should be interpreted cautiously when considering their relevance to human contexts. 

 The variation in intervention methods and the way professionals interact with children significantly influences success and adherence. Aspects such as session duration and exercise intensity can be adjusted, with evidence that even short, intense sessions produce significant results. However, a lack of incentives or inadequate follow-up can lead to dropout, as observed in some studies. In addition to immediate benefits, the positive effects of physical activity extend to daily life, including improvements in school performance and social behavior, reinforcing the importance of incorporating physical activity into the treatment of children with ADHD. Playful and interactive interventions, conducted with appropriate professional support, optimize cognitive and behavioral development and provide a positive social environment with lasting impacts on children and their families. 

 The results indicate that participation in physical activity and exercise enhances cognitive function. However, a limitation of the research is the inability to identify the optimal type of exercise due to the wide variation in the intervention protocols. Furthermore, despite the cognitive improvements observed, it is difficult to determine which specific cognitive domains are most positively affected by physical activity, given the considerable diversity of measurement instruments employed. In this context, the unique physiological and cognitive demands of different types of physical activity could lead to various adaptations in brain structure and function. 

 It is important to note that this research was limited to specific databases and publications in English, which may have excluded relevant evidence available in other languages. Another significant limitation is that the methodological quality of the included studies was not assessed, which diminishes the strength of the conclusions drawn. Additionally, variability in methods, samples, and outcomes among the analyzed studies hinders a more comprehensive synthesis of the findings. A further limitation lies in the extrapolation of findings from animal models to human contexts. While animal studies provide valuable insights into the neurobiological mechanisms linking physical activity and cognition, species differences restrict the direct application of these results to humans. Therefore, the findings should be interpreted with caution when applied to human situations. 

 The selected studies varied in intervention duration, ranging from 8 to 12 weeks. For future research, it is recommended to follow national or international guidelines regarding the frequency and intensity of physical activity and exercise. It is also essential to consider children’s preferences when designing training sessions. Additionally, we suggest implementing a comprehensive cognitive test battery that assesses multiple cognitive domains. Evaluating physical capabilities and, when possible, collecting biological samples, such as blood, to analyze BDNF levels and other potential biomarkers, would also be valuable. This approach may help determine whether these variables are related to cognition in children with ADHD. 

## Data Availability

No new data were generated or analyzed in this study.
